# The Treatment of Minor Epilepsy

**Published:** 1900-01-13

**Authors:** 


### THE TREATMENT OF MINOR EPILEPSY.

In a clinical lecture recently delivered at the National Hospital for Epilepsy and Paralysis, Sir William Gowers[1] pointed out the great variety of forms which attacks of minor epilepsy may take, and showed that, although in the most typical cases of this disease loss of consciousness is often the only symptom, an attack of true minor epilepsy may occur without any such loss. "The statement which is often made that loss of consciousness is an invariable and essential feature of epilepsy is," he says, "certainly erroneous. There is usually, however, some obscuration of consciousness which may have various forms. One of the most frequent is a sense of strangeness. The patient feels for the moment as if in unfamiliar surroundings." As to the sensations which attend these attacks of minor epilepsy their variety is very great, and the same is true in regard to the after states, in some of which actions may be performed which may lead to many complications. Momentary as may have been the attack, normal consciousness is often regained but slowly, and when recovery is thus deliberate certain actions may be performed automatically during this state of imperfect recognition of surroundings, as a result of some dominant idea. A very common thing is for the patient to begin to take off his clothes, and as this action may follow an attack so slight as not to have been noticed, very embarrassing results may follow. Other patients, again, have a tendency during this the post-epileptic condition to put into their pockets whatever is within reach of their hands, and this may give rise to very inconvenient suspicions. We may add, however, that these actions do not always seem to be done under the influence of a dominant idea, but rather as the result of an automatic or unthinking response to surrounding conditions, as, for example, in the case given of a carman who, after an attack of minor epilepsy which was not sufficient to interfere with his seat on the box, went on driving through the streets of London quite safely but in the wrong direction; and another case in which a lady, after an attack which took place while she was at the piano, went on playing elaborate music quite correctly until she "came to herself" and stopped, unable to understand what she was doing. When we come to treatment, we do not find much beyond the advice to try things carefully and see what is their effect. "Unfortunately, we are not able to discern what will happen in any given case. In the case of all drugs and all forms of the disease it is most difficult to discern 'indications' for treatment; conditions which seem to have determined success in one case are found in others to have no value. We can only try and see the result." Although bromide acts in minor epilepsy with much less constancy than in the severe attacks, still it is not without effect, and it may sometimes stop the slight as well as the severe attacks, and may also stop the attacks in a patient who has only the minor form. Then there is zinc, the oxide of which is perhaps the best preparation; and among agents which are clearly successful in some cases of minor epilepsy are nitroglycerine, given three times a day, and the hydrobromate of hyoscine, belladonna, and Indian hemp. Strychnine also is occasionally of service, especially in combination with nitroglycerine. It is to be remembered that when treatment is to be discontinued this must be done by gradual diminution and not by sudden cessation, and also that patients who have had severe attacks are sure to have an early recurrence if, after discontinuance of treatment, they still have occasional minor attacks, however slight.

[1] Brit. Med. Jour., Jan. 6.

